# State equity participation and financing constraints of private enterprises in China: Based on the competitive pressure perspective

**DOI:** 10.1371/journal.pone.0292817

**Published:** 2023-11-15

**Authors:** Qian Xiao

**Affiliations:** College of Business, Huaihua University, Huaihua, Hunan, China; Jiangsu University, CHINA

## Abstract

This study examines the impact of state participation on the alleviation of financing constraints faced by private Chinese enterprises. The analysis is based on data collected from a sample of 2,256 private Chinese enterprises surveyed by the World Bank in 2011 and 2013, and a sample of 3,197 listed enterprises in China from 2009 to 2020. The empirical findings demonstrate that (1) State equity participation can effectively alleviate the financial constraints faced by enterprises, and its approach is to alleviate financing constraints by lessening the competitive pressure on mixed-equity enterprises. (2) Anxiety related to survival and development that arises from competitive pressures compels private enterprises to seek competitive advantages through new investments. Consequently, private enterprises’ demand for external capital has increased. However, the conversion of capital requirements into external financing applications has become increasingly challenging due to deficiencies in enterprises’ internal cognitive processes regarding financial information. (3) Heterogeneity analysis reveals that state equity participation has a more pronounced impact on alleviating the financing constraints faced by emerging, non-group, and non-listed enterprises.

## 1. Introduction

In recent decades, the rapid development of China’s economy has been supported by the important contributions of private enterprises in promoting employment, technological innovation, and financial tax revenue [1]. In most global economies, there has been a gradual improvement in both the macro policy environment and business conditions. This has resulted in more opportunities for private enterprises to enhance their quality of development [2]. However, private enterprises still face significant challenges in accessing affordable financing, which is a major obstacle to their growth and development [3]. Since 2020, the COVID-19 pandemic has decelerated China’s economic growth and increased financing challenges for private enterprises [4]. Previous studies have demonstrated that the presence of financial constraints adversely affects the digital transformation and green innovation capacities of private enterprises while concurrently amplifying their carbon dioxide emission and energy intensities [5–8]. Hence, the effective alleviation of financial constraints for private enterprises has emerged as a crucial concern for ensuring the high-quality advancement of the private sector.

In 2015, the State Council of China proposed promoting state-owned capital to acquire equity holdings in non-state-owned enterprises through various means. Consequently, state-owned capital is increasingly engaging in private enterprises through equity participation. State participation in private enterprises is prevalent in economies where public ownership is predominant. Among the various types of intervention, direct state equity participation in private enterprises has a more extensive influence on their overall growth. The government not only impacts the growth plan and benefit-sharing of private enterprises based on equity but also offers enterprises unique political relations and internal and external resources. The question of whether the participation of state equity in private enterprises for the purpose of implementing hybrid ownership reforms has a positive impact on the long-term growth of these enterprises has garnered significant attention in recent years [9,10]. Competitive pressure on enterprises causes survival and development concerns caused by an increasingly competitive marketplace. When enterprises face intense competitive pressure, they strive to acquire a unique competitive edge through new investments, such as research and development (R&D) or the introduction of innovative technologies. Consequently, the heightened competitive pressure in the external market amplifies an enterprise’s requirement for external financial resources.

Investigating the constraints faced by enterprises in obtaining funding initially emerged from the Modigliani (MM) hypothesis in the 1950s. After extensive theoretical expansion and development, Fazzari et al. established a widely acknowledged definition of financing constraints [11]. These constraints arise from the imperfect nature of the capital market, resulting in the absence of a perfect substitution relationship between internal and external financing for enterprises. Consequently, external financing incurs a financing premium, prompting enterprises to rely heavily on internal funds for investments. This dynamic gives rise to financing constraints experienced by enterprises. Some scholars claim that the underlying reason for constraints in enterprise financing may be the response of enterprise finance to fluctuations in the macroeconomic landscape or alterations in macroeconomic policy. Illustrative examples include economic operating circumstances [12,13], monetary policy [14], banks’ market power [15], and financial technology [16]. There is a body of research indicating that corporate funding limits may be attributed to businesses’ specific factor endowments. For instance, Almeida and Campello reveal that enterprises possessing greater tangible assets may have enhanced access to external financing [17]. Private- and foreign-owned enterprises are more susceptible to financial limitations than state-owned and collective enterprises [18]. Moreover, it should be noted that smaller non-state enterprises are particularly susceptible to customer concentration. This concentration of consumers results in amplified bargaining power for downstream customers during supplier-customer interactions. Consequently, this dynamic exacerbates the financial restrictions faced by upstream organizations [19].

Combining the above literature, it can be seen that most studies on the factors affecting the financing constraints of private enterprises focus on the external policy and financial environments of enterprises. Although there are some studies on the internal nature of enterprises, their theories need to be further improved. Financing constraints have always been a key topic of researchers’ attention, but there are still some shortcomings to be addressed: (1) Influenced by the availability of data, most studies have focused on the data of listed enterprises, or the raw data obtained from research in specific regions, and studies on national unlisted enterprises still need to be supplemented. (2) Some studies consider enterprise competition as an important factor affecting financing constraints, but the theory of its functioning mechanism has not yet been fully formed and still needs to be further improved. (3) The impact of state participation on the external financing constraints of private enterprises is not yet conclusive, and few studies have focused on the political affiliation and competitive dynamics of private enterprises. Therefore, studying the impact of state participation and private enterprises’ internal competitive pressure on their financing constraints both answers whether private enterprises can take the initiative in the capital market and is a useful suggestion for government departments and financial institutions to effectively help private enterprises in financing.

The contributions of this paper are as follows: (1) Taking private enterprises as the research object, we explore the impact of state-owned capital participation on corporate financing constraints from the perspective of state-owned capital participation, enriching the relevant literature on the impact of state-owned participation on the financing constraints of private enterprises. (2) We explore the mechanism of how competitive pressure derived from market competition behavior impact the financing constraints of private enterprises and explore whether state-owned equity participation in private enterprises can alleviate internal competitive pressure and financing constraints. (3) By adding data on small- and medium-sized private enterprises, we verify whether the effect of state-owned equity participation on private enterprises’ external financing constraints is universal.

The rest of the paper is structured as follows: the second section presents the theoretical analysis and research hypotheses; the third section describes the data sources and variable selection; the fourth and fifth sections present the empirical analyses, including the results of the benchmark regressions, mechanism analyses, heterogeneity analyses, and robustness tests; the sixth section presents the discussion; and the seventh section presents the conclusions and policy recommendations.

## 2. Theoretical analysis and research hypotheses

### 2.1 State participation and financial constraints

The relationship between political affiliation and the financing constraints of private enterprises has been a popular topic of academic concern, and most scholars agree that political affiliation can alleviate enterprises’ financing constraints [20–22]. For example, Chan et al. argue that enterprises with political links do not have financing constraints, whereas enterprises without political links have significant financing constraints, and that private enterprises without political links are more constrained than state-owned enterprises without political links [23]. Houston et al. found that bank loan costs were significantly lower for enterprises with politically connected board members [24]. However, other scholars hold the opposite view, arguing that political affiliation increases the cost of debt financing for enterprises [25], thereby making it more difficult for them to raise capital. State equity participation is a unique form of political affiliation [26]. By establishing a relatively stable symbiotic relationship with the government through state shareholders, private enterprises can mitigate the negative impact of unfavorable factors such as credit discrimination and strive for relevant benefits and preferential treatment for private enterprises, thus alleviating their financing constraints [27]. For example, Boubakri and Saffar argued that state participation could bring more bank debt financing to private enterprises, suggesting that private enterprises benefit from the soft budget constraints imposed by state participation [28].

Political affiliation is an intangible asset of private enterprises, an important signal for private enterprises to differentiate themselves from competitors and provides more channels for private enterprises to source their endowments. The signaling mechanism of state equity participation alleviates information asymmetry between private enterprises and financial institutions, which takes ownership of state equity by private enterprises as an important signal of solvency. Thus, state equity participation can alleviate the unfavorable state of enterprises in a competitive market and does not create strong competitive pressure in the face of competitors’ behaviors due to confidence in their products or enterprises. Accordingly, it can be argued that state equity participation alleviates financing constraints by reducing market competition and competitive pressures. Therefore, this study proposes hypotheses H1 and H1a:

H1: State equity participation eases the financing constraints of private enterprises.
H1a: State equity participation eases the competitive pressure on private enterprises.

Discussing further the mechanism of competitive inertia generated by state equity participation, private enterprises with state equity participation face less competitive pressure to expand or engage in other competitive behaviors, and confidence in their endowment further reduces the need for external finance. Therefore, it can be argued that state equity participation also reduces the demand for external funds and, in turn, reduces the likelihood that private enterprises will request funds from external institutions. Accordingly, H1b is formulated as follows:

H1b: State equity participation reduces private enterprises’ demand for external financing and the likelihood of external financing applications.

### 2.2 Competitive pressure and financing constraints

Market competition has been identified as one of the factors affecting the financing constraints of enterprises. Mackay and Phillips found that banks consider the degree of competition in the industry in which an enterprise operates when contracting a loan with the enterprise [29]. Enterprises’ capital requirements have increased in response to the predatory risks associated with market competition. However, banks and investors consider these default risks and accordingly adjust the price of financing contracts, making enterprises less capable of raising capital [30]. Market competition exacerbates the risk of unfair dealings between enterprises and investors, ultimately leading to constraints on enterprises’ external financing [31]. Povel and Raith argue that market competition increases enterprises’ risk of bankruptcy, which in turn increases the uncertainty of future earnings or going concerns, leading banks, or lenders to take a negative view of an enterprise’s solvency and exacerbate financing constraints [32]. At the same time, financing constraints can adversely affect market competition for an enterprise’s products. Desai et al. found that multinational enterprises with fewer financing constraints are better able to seize market opportunities and expand and capture markets more quickly than local enterprises [33]. In conclusion, competitive pressures within enterprises lead to an increase in cash flow requirements, but at the same time, the risks faced by enterprises also increase, exacerbating their financing constraints under dual pressures.

Competitive pressure is generated by business operators based on the behavior of their competitors in the market. Competitive pressure manifests itself in the fear of internal managers’ market capture due to a shift in competitors’ strategies or organizational change. Similarly, according to the connotation of competitive pressure, it can also be used to indicate the size of an enterprise’s market power because the leading enterprise in the market does not pay special attention to the behavior of its competitors, while the subordinate enterprise will focus on the behavior of its competitors. Therefore, internal competitive pressure derived from the external market’s competitive environment is one of the most important factors affecting private enterprises’ business decisions. The impact of competitive pressure on business operations is multifaceted, focusing on the potential investments that enterprises can make in response to competitors’ strategic shifts. Potential investments further evolve into external demand for financing, but formal or informal financial institutions consider the competitive status of enterprises in the market when making decisions. This is demonstrated by the fact that financial institutions perceive that enterprises with market power have stronger potential profitability and, therefore, stronger debt-servicing ability, while enterprises with weaker market power are less profitable, and lending to weaker enterprises is too risky and prone to evolve into bad debts for financial institutions, thus further deepening the financing constraints of enterprises with weaker market power. Thus, competitive pressure increases private enterprises’ financing constraints by increasing their external demand for funds. This study proposes the H2 and H2a hypotheses:

H2: Competitive pressure elevates the financing constraints of private enterprises.
H2a: Competitive pressure raises the external financing demand of private enterprises.

Private enterprises’ external demand for financing, which is heightened by competitive pressures, is externalized through their operations into claims for financing. In turn, private enterprises make financial claims to formal financial institutions, such as banks, informal financial institutions, or upstream and downstream enterprises in the supply chain. Therefore, it is believed that competitive pressure ultimately enhances the possibility of the occurrence of private enterprises’ external financial claims, and the hypothesis is formulated accordingly:

H2b: Competitive pressure enhances the incidence of private enterprises’ external financial applications.

**Fig 1:**
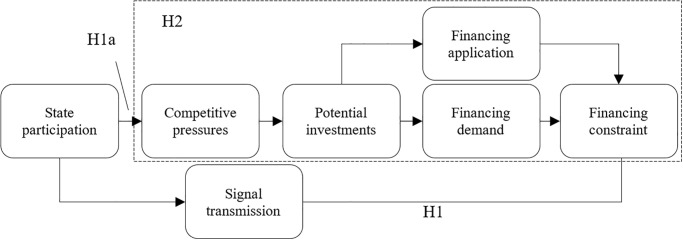
Theoretical paths and research hypotheses.

The theoretical path and assumptions of this study are illustrated in Fig 1.

## 3 Data source and variable description

### 3.1 Data source

The data used in this study are enterprise-level data from the World Bank Enterprise Surveys for China in the period 2011–2013 (http://www.enterprisesurveys.org), which involve a large number of surveyed enterprises and a large number of variables and provide a better source of data for the study of China’s economic problems. Samples of state-owned enterprises in the database and those with missing relevant data are excluded to obtain a total sample of 2,256 private enterprises. Although the database is relatively old, it contains indicators of competitive pressure, financing needs and financing applications of private enterprises, and is therefore more suitable for use in addressing the issues of concern in this paper. Moreover, to extend the generality and generalizability of the core findings, this paper uses data from 3,197 listed firms in China from 2009–2020 for robustness testing.

### 3.2 Variable description

**Fig 2:**
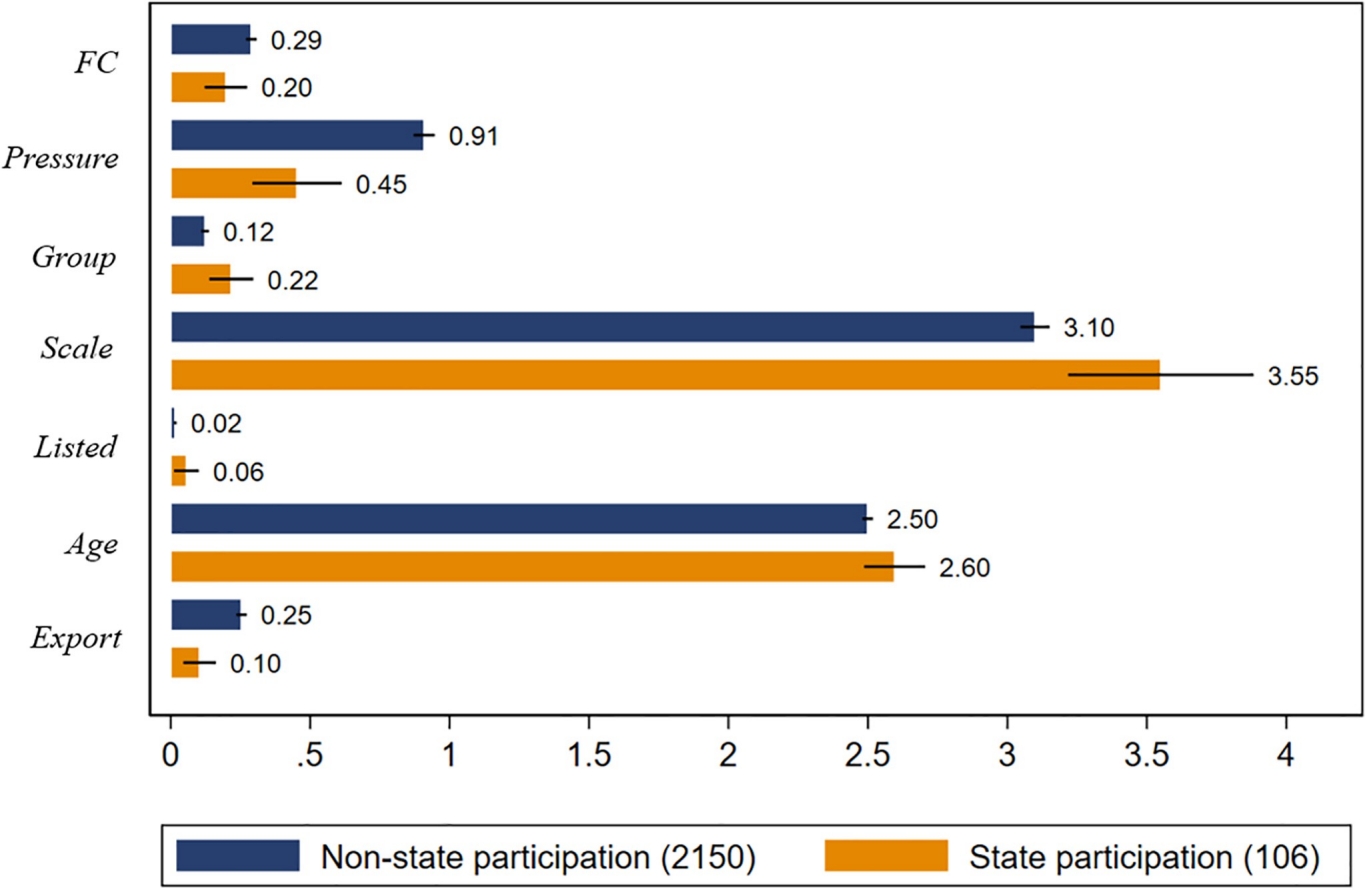
Descriptive statistics of variables between groups.

1. Explained variable: financing constraints. Enterprises facing financing constraints were defined as 1; otherwise, 0. A financing constraint occurs when an enterprise has a need for funds, but this need is not satisfied. Based on related research, this study measured enterprises’ financing constraints from two perspectives [34]. Firstly, with regard to financial demand, the existence of financial demand was determined by the responses of the surveyed enterprises to the questions "Did this establishment apply for any loans or lines of credit?" What was the main reason this establishment did not apply for any line of credit or loans?—No need for a loan establishment; had sufficient capital. If an enterprise answers "no" to the first question but "yes" to the second question, it is assumed that the enterprise does not have a need for capital and thus does not have a financing constraint. If an enterprise answers "yes" to the first question but "no" to the second question, it is considered to have a need for capital, but it has not applied for credit for other reasons and is therefore considered to be facing a financing constraint. Secondly, with regard to whether the enterprise’s need for funds has been satisfied, it is judged by the surveyed enterprises’ responses to the questions "Did this establishment apply for any loans or lines of credit?" At this time, does this establishment have a line of credit or loan from a financial institution?". If the enterprise answers "yes" to both of these questions, it means that the enterprise’s financial needs are met and, therefore, the enterprise does not face a financing constraint.

2. Explanatory variables: (1) State participation: enterprises with state participation are assigned a value of 1, and enterprises without state participation are assigned a value of 0. The proportion of state participation was used as a proxy variable in the robustness test. (2) Competitive pressure: based on the enterprise’s response to the competitive pressure question, the options for this question were handicap-free, minor obstacles, general obstacles, major obstacles, and serious obstacles, with values of 0, 1, 2, 3, and 4.

3. Control variables: (1) Enterprise size, expressed in terms of the number of full-time employees in the enterprise and taking a natural logarithm [35]. It is generally assumed that the larger the enterprise, the lower the likelihood of financing constraints. (2) Enterprise age. We use 2011 minus the year in which the enterprise was established to obtain its age and take the natural logarithm. Enterprises with more years of establishment generally form better social relationships with financial institutions and supply chain enterprises and are more likely to obtain funds from external sources. (3) Is an enterprise listed? If the enterprise is listed, it is 1; if the enterprise is unlisted, it is 0. Listed enterprises are generally considered to have greater market power and, therefore, a lower likelihood of financing constraints [36]. (4) Is an enterprise a part of a large enterprise? If an enterprise is a large enterprise, it is 1. Large enterprises are more likely to receive financial support from the parent enterprise and, therefore, have a lower possibility of financing constraints. (5) Whether the enterprise’s products are exported. Enterprises’ products are exported is 1, not exported and 0. It is generally believed that listed enterprises have a strong market power; therefore, the possibility of financing constraints is low. (6) To avoid the possibility of bias caused by the heterogeneity of cities or industries in the model estimation, city dummy variables (City FE) and industry dummy variables (Industry FE) are introduced [37]. Descriptive statistics for the key variables are shown in Table 1, and descriptive statistics of the variables between the groups are shown in Fig 2.

**Table 1:** Descriptive statistics.

Variable type	Variable	Abbreviate	Obs.	Mean	Std. Dev.	Min	Max
Explained variable	Financing constraint	FC	2,256	0.2850	0.4515	0	1
	Financing convenience	Convenience	2,256	0.8285	0.8771	0	4
	Financing demand	Demand	2,256	0.6219	0.4850	0	1
	Financing application	Application	2,256	0.3524	0.4778	0	1
Explanatory variable	State participation	SP	2,256	0.0470	0.2117	0	1
	Percentage of state participation	PSP	2,256	3.5231	16.6522	0	95
	Competitive pressures	Pressure	2,256	0.8874	0.8948	0	4
Control variable	Enterprise age	Age	2,256	2.5036	0.4525	0	4.8363
	Listed enterprises	Listed	2,256	0.0182	0.1336	0	1
	Enterprise scale	Scale	2,256	3.1202	1.2640	0	9.6550
	Group enterprises	Group	2,256	0.1277	0.3338	0	1
	Exporting enterprises	Export	2,256	0.2469	0.4313	0	1

## 4 Empirical analysis

### 4.1 Benchmark regression results

To verify the hypotheses, Probit and OLS models were constructed. The benchmark regression results are presented in Table 2. Columns 1–2 report the regression results without controlling for the control variables and fixed effects, columns 3–4 report the regression results controlling for the control variables, and columns 5–6 report the regression results controlling for the control variables and fixed effects. These results indicate that state equity participation significantly alleviates financing constraints. Columns 7–8 report the results of the robustness test of the OLS model, which remains significantly positive.

**Table 2:** Benchmark regression results.

Variables	(1) Probit	(2) Probit	(3) Probit	(4) Probit	(5) Probit	(6) Probit	(7) OLS	(8) OLS
	FC	FC	FC	FC	FC	FC	FC	FC
SP	-0.2930**		-0.4135***		-0.3451**		-0.0785*	
	(0.1420)		(0.1589)		(0.1689)		(0.0416)	
PSP		-0.0039**		-0.0061***		-0.0056***		-0.0014***
		(0.0018)		(0.0020)		(0.0021)		(0.0005)
Pressure					0.1534***	0.1518***	0.0403***	0.0397***
					(0.0389)	(0.0389)	(0.0106)	(0.0106)
Group					-0.1823*	-0.1870*	-0.0501*	-0.0511*
					(0.1092)	(0.1091)	(0.0270)	(0.0269)
Scale					-0.1323***	-0.1327***	-0.0334***	-0.0331***
					(0.0307)	(0.0307)	(0.0078)	(0.0077)
Listed					-0.0506	-0.0311	0.0041	0.0062
					(0.3132)	(0.3143)	(0.0552)	(0.0559)
Age					-0.1984***	-0.1979***	-0.0505***	-0.0502***
					(0.0705)	(0.0707)	(0.0186)	(0.0186)
Export					-0.4099***	-0.4154***	-0.0973***	-0.0994***
					(0.0875)	(0.0876)	(0.0221)	(0.0221)
Controls	No	No	No	No	Yes	Yes	Yes	Yes
City FE	No	No	Yes	Yes	Yes	Yes	Yes	Yes
Industry FE	No	No	Yes	Yes	Yes	Yes	Yes	Yes
*N*	2,256	2,256	2,194	2,194	2,194	2,194	2,256	2,256
*R-squared*							0.214	0.215
*Pseudo R2*	0.002	0.002	0.154	0.155	0.192	0.193		

Note: Robust standard errors are in parentheses.

*, **, and *** represent significance at the 10%, 5%, and 1% levels, respectively.

### 4.2 Mechanisms analysis results

#### 4.2.1 Alleviating competitive pressures

Theoretical analyses suggest that state equity participation eases corporate financing constraints by reducing the competitive pressures on enterprises and thereby easing corporate financing constraints. Columns 1–4 in Table 3 report the regression results in which the explained variable is competitive pressure, indicating that state equity participation significantly reduces competitive pressure on enterprises. Columns 5–6 report the results of the regressions where the explanatory variable is competitive pressure, and the explained variable is financing constraints, indicating that competitive pressure significantly increases enterprises’ financing constraints. These results confirm that state equity participation eases enterprises’ financing constraints by reducing the competitive pressure.

**Table 3:** Mechanism test results I.

Variables	(1) OLS	(2) Order-Probit	(3) OLS	(4) Order-Probit	(5) OLS	(6) Probit
	Pressure	Pressure	Pressure	Pressure	FC	FC
SP	-0.2655***	-0.5428***				
	(0.0808)	(0.1504)				
PSP			-0.0036***	-0.0075***		
			(0.0010)	(0.0019)		
Pressure					0.0422***	0.1631***
					(0.0106)	(0.0390)
Controls	Yes	Yes	Yes	Yes	Yes	Yes
City FE	Yes	Yes	Yes	Yes	Yes	Yes
Industry FE	Yes	Yes	Yes	Yes	Yes	Yes
*N*	2,256	2,256	2,256	2,256	2,256	2,194
R-squared	0.223		0.224		0.213	
Pseudo R2		0.106		0.106		0.190

To analyze the competitive advantage of state participation in more depth, this study considers the obstacles faced by enterprises in the court and tax systems as explained variables. The results in columns 1–4 of Table 4 show that state equity participation significantly reduces obstacles from court and tax systems. In turn, this creates a unique competitive advantage for the enterprise and eases competitive pressure.

**Table 4:** Mechanism test results II.

Variables	(1) OLS	(2) Order-Probit	(3) OLS	(4) Order-Probit
	Obstacles from the court system	Obstacles from the court system	Obstacles from the tax system	Obstacles from the tax system
SP	-0.3645***	-0.6460***	-0.1248*	-0.2352*
	(0.0618)	(0.1096)	(0.0705)	(0.1329)
Controls	Yes	Yes	Yes	Yes
City FE	Yes	Yes	Yes	Yes
Industry FE	Yes	Yes	Yes	Yes
*N*	2,236	2,236	2,253	2,253
R-squared	0.178		0.455	
Pseudo R2		0.094		0.210

#### 4.2.2 Reducing financing demand and financing application

This section deconstructs financing constraints into financing needs and financing applications, based on which it attempts to verify the role of state equity participation. According to columns 1–4 in Table 5, state equity participation significantly reduces enterprises’ financing demands and financing applications, confirming the important role of state-owned equity in enriching the internal capital of enterprises. However, the empirical results show that competitive pressure on enterprises enhances the demand for financing, verifying that competitive pressure encourages enterprises to enhance the potential demand for investment to gain a competitive advantage. However, competitive pressure from enterprises does not increase financing applications, suggesting that financing needs stemming from competitive pressure are less likely to be converted into financing applications due to information asymmetry in private enterprises’ access to formal finance.

**Table 5:** Mechanism test results III.

Variables	(1)	(2)	(3)	(4)
	Demand	Demand	Application	Application
SP	-0.5412***		-0.6518***	
	(0.1390)		(0.1665)	
PSP		-0.0081***		-0.0097***
		(0.0018)		(0.0023)
Pressure	0.1070***	0.1052***	0.0069	0.0066
	(0.0376)	(0.0376)	(0.0376)	(0.0376)
Controls	Yes	Yes	Yes	Yes
City FE	Yes	Yes	Yes	Yes
Industry FE	Yes	Yes	Yes	Yes
*N*	2,246	2,246	2,250	2,250
Pseudo R2	0.1436	0.1500	0.2108	0.2102

### 4.3 Heterogeneity analysis results

Heterogeneity tests were conducted based on the cross-multiplication terms between the variables. The results in column 1 of Table 6 indicate that the financing constraint alleviation effect of state equity participation is more significant in younger enterprises than in older ones. The results in columns 2–3 indicate that the financing constraint mitigation effect of state equity participation is more significant among non-group and unlisted enterprises. This may be because older enterprises, group enterprises, and listed enterprises have access to a richer range of sources of financing and are, therefore, not dependent on state equity participation.

**Table 6:** Results of heterogeneity analysis.

Variables	(1) Probit	(2) Probit	(3) Probit
	FC	FC	FC
SP	-0.5361***	-0.4564**	-0.4441**
	(0.2015)	(0.1822)	(0.1754)
SP × Age	0.0146*		
	(0.0084)		
SP × Group		0.7865*	
		(0.4185)	
SP × Listed			1.7755**
			(0.6940)
Controls	Yes	Yes	Yes
City FE	Yes	Yes	Yes
Industry FE	Yes	Yes	Yes
*N*	2,194	2,194	2,194
*Pseudo R2*	0.1918	0.1926	0.1938

## 5. Robustness test

### 5.1 Endogeneity test results

The previous section carried out the corresponding empirical research on the relevant assumptions but still cannot reject the endogeneity of the statistical results due to the problem of bias. The Chinese government, in the selection of private enterprises for equity investment, will fully consider the current situation of private enterprises and business prospects. Therefore, there is a possibility that the local government’s adverse selection leads to the possibility of having state equity participation due to the enterprise’s endogenous capital adequacy, and this possibility will lead to endogeneity of the econometric model due to reverse causality; to alleviate the endogeneity of the model, this study adopts the instrumental variable method to test the model. The average values of the proportion of state equity participation and the competitive pressure of other enterprises in the same industry were chosen as instrumental variables for the instrumental regression. The regression results are shown in Table 7. Columns 1 and 4 report the first-stage regression results, which show that state equity participation and competitive pressure are significantly negatively related to the average of other enterprises in the same industry and that the instrumental variables passed the unidentifiable and weak instrumental variable tests in both models. Columns 2, 3, and 5 report the results of the second-stage regression, which show that after mitigating the endogeneity problem using instrumental variables, the share of state equity participation still significantly affects enterprises’ financing constraints and competitive pressures, while enterprises’ competitive pressures still significantly and positively affect enterprises’ financing constraints. In summary, the results of previous studies remain robust after instrumental variables are used to mitigate the endogeneity problem due to reverse causation.

**Table 7:** Results of endogeneity test.

	(1) First stage	(2)	(3)	(4) First stage	(5)
	SP	FC	Pressure	Pressure	FC
SP		-0.3818*	-0.7841***		0.1914**
		(0.1863)	(0.1873)		(0.0748)
IV_SP	-0.9230***				
	(0.0109)				
IV_PP				-28.8435***	
				(0.9338)	
Controls	Yes	Yes	Yes	Yes	Yes
City FE	Yes	Yes	Yes	Yes	Yes
Industry FE	Yes	Yes	Yes	Yes	Yes
*N*	2,194	2194	2,194	2,194	2,194
F-statistic		7654.91	7699.43		414.33

### 5.2 The result of changing the calculation method of the explained variable

To avoid statistical bias in the calculation method of financing constraints in the conclusions of this study, the explained variable was changed to financing convenience. The empirical results in Table 8 indicate that the benchmark empirical results remain significantly negative after changing the calculation method of the explained variable.

**Table 8:** Results of robustness test.

Variables	(1) OLS	(2) Order-Probit	(3) OLS	(4) Order-Probit
	Convenience	Convenience	Convenience	Convenience
SP	-0.1983***	-0.4271***		
	(0.0757)	(0.1494)		
PSP			-0.0032***	-0.0074***
			(0.0009)	(0.0019)
Pressure	0.0886***	0.1536***	0.0875***	0.1524***
	(0.0233)	(0.0343)	(0.0233)	(0.0343)
Controls	Yes	Yes	Yes	Yes
City FE	Yes	Yes	Yes	Yes
Industry FE	Yes	Yes	Yes	Yes
*N*	2,256	2,256	2,256	2,256
*R-squared*	0.272		0.273	
*Pseudo R2*		0.1321		0.1334

### 5.3 Using data of Chinese main board-listed enterprises

To extend the period of the data and make the research findings more comprehensive and general, this study used the data of Chinese main board-listed enterprises from 2009 to 2020 as samples in the robustness test. This study refers to Whited-Wu’s study to calculate the financing constraints of enterprises [38]. We took the proportion of enterprise state-owned participation and enterprise state-owned participation as explanatory variables; the control variables included the age of the enterprise, the number of employees of the enterprise, the size of the director, the number of assets of the enterprise, the value of Tobin’s Q, the intensity of R&D, and the rate of return on assets. The empirical results in Table 9 show that the benchmark empirical results remain significantly negative after changing the use of listed enterprise data.

**Table 9:** Using data from Chinese main board listed enterprises.

Variables	(1)	(2)	(3)	(4)
	Financing constraints	Financing constraints	Financing constraints	Financing constraints
SP	-1.6994**	-1.7008**		
	(0.8290)	(0.8639)		
PSP			-0.1132***	-0.1195***
			(0.0283)	(0.0295)
Controls	Yes	Yes	Yes	Yes
Year FE	Yes	Yes	Yes	Yes
Enterprise FE	Yes	Yes	Yes	Yes
*N*	19,978	19,508	19,978	19,508
*R-squared*	0.101	0.101	0.101	0.101

### 5.4 Results of propensity score matching (PSM)

**Fig 3:**
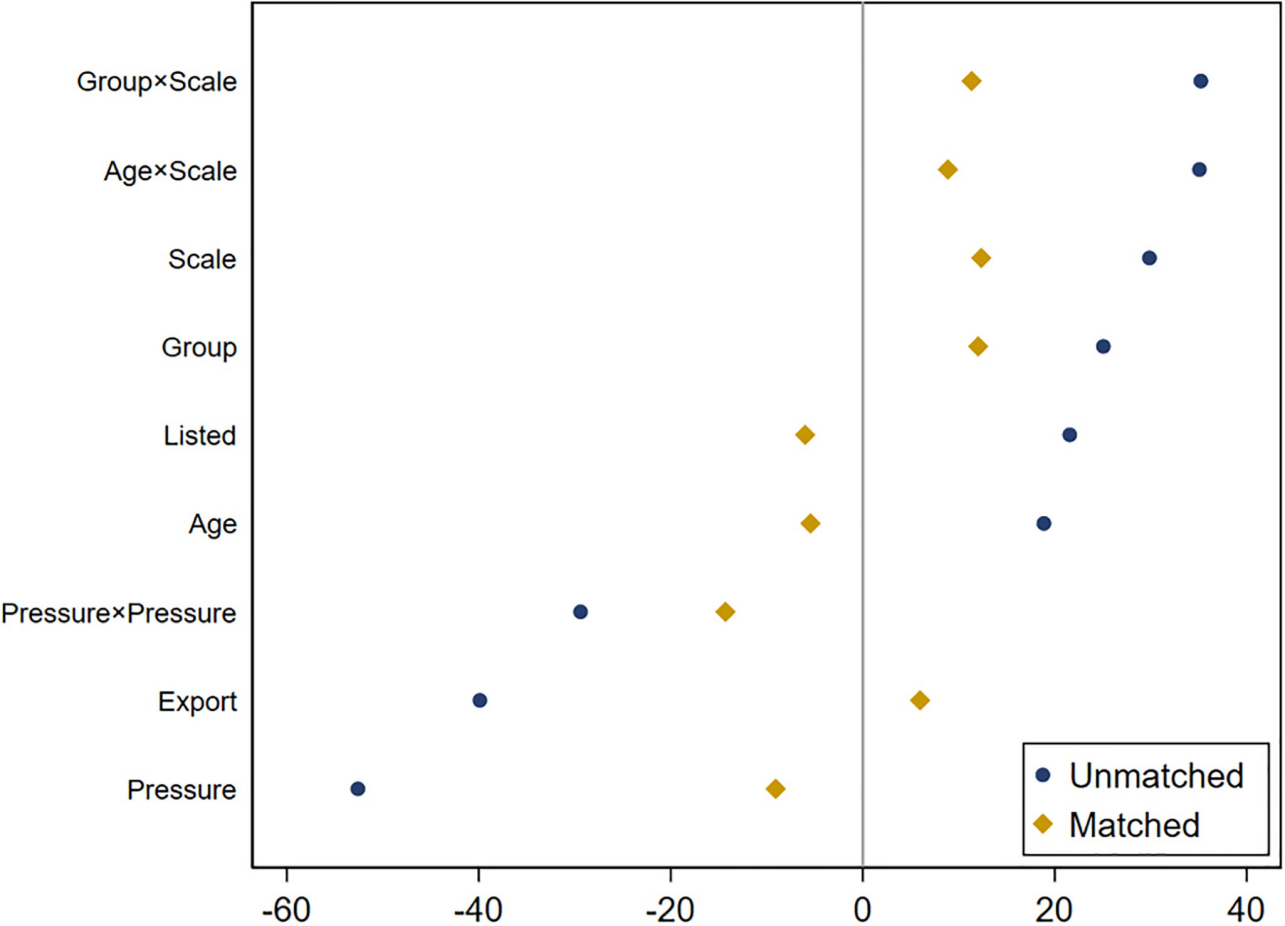
Results of the balance test. Note: After propensity score matching, the difference between state-participating and non-state-participating enterprises in the covariates was less than 20%.

Avoiding the characteristics of state-participating enterprises differing considerably from those of non-state-participating enterprises could lead to biased statistical results. This study selects covariates based on *psestimates* and matches the two groups of enterprises based on the covariates, thereby excluding enterprises with large differences. The empirical results for the retained matched sample are shown in Table 10, indicating that the benchmark empirical results remain robust based on reduced between-group differences. Fig 3 shows the results of the balance test, indicating that the matched samples satisfied the balance.

**Table 10:** Results of propensity score matching (PSM).

Variables	(1) Probit	(2) Probit	(3) OLS	(4) OLS
	FC	FC	FC	FC
SP	-0.4219**		-0.1129**	
	(0.1904)		(0.0505)	
PSP		-0.0068***		-0.0019***
		(0.0024)		(0.0006)
Pressure	0.2983***	0.2958***	0.0851***	0.0837***
	(0.0646)	(0.0647)	(0.0181)	(0.0181)
Controls	Yes	Yes	Yes	Yes
City FE	Yes	Yes	Yes	Yes
Industry FE	Yes	Yes	Yes	Yes
*N*	1,123	1,123	1,183	1,183
*R-squared*			0.286	0.288
*Pseudo R2*	0.2334	0.2355		

## 6. Discussion

Since 2020, the COVID-19 Pandemic has caused China’s economy to slow down, and financing China’s private companies has become more difficult. Severe financing constraints limit firms’ performance in technological innovation, the introduction of emerging technologies, and CO2 emissions reduction. This study focused on the impact of state-equity participation on the financing constraints of private enterprises in China. As a public sector-based economy, the Chinese government has influenced the development and strategy formulation of private enterprises in various ways. Additionally, as an important form of state intervention in private enterprises, state equity participation has a significant impact on private enterprise development. Reducing private-sector financing constraints through equity participation may be an important policy direction for governments. This study is important for policymakers and theoretical researchers. To formulate more effective policies to alleviate the financing constraints of private enterprises, they must identify the facts and mechanisms through which state-owned equity participation affects the financing constraints of private enterprises.

The political affiliation and credit endorsement provided by state-owned equity participation provide private firms with access to external financing. Overall, the results indicate that state-owned equity participation significantly alleviates private firms’ financing constraints. This finding is similar to that reported by Li et al [39]. To explore the competitive advantages arising from state ownership, this study also explored the barriers to private enterprises arising from judicial and tax systems. It is further confirmed that state equity participation significantly reduces the barriers to private enterprises created by the judicial and tax systems. It confirms the hypothesis of political affiliation advantage proposed in the research results of Chan et al [40]. Based on the results of Bai et al. [41], this study conducted a robustness test using data from the main Chinese board-listed companies, and the results show that the effect of state-owned equity participation on the financing constraints of private firms still holds in a large sample of listed companies.

Chen and Guariglia showed that older and group p-type firms are more likely to obtain external funding from other sources [18]. Therefore, this study argues that state-owned equity participation may only have an impact on young and non-group firms and confirms this view in the heterogeneity test.

## 7. Conclusions

Our overall results can be summarized as follows. First, state equity participation significantly alleviates private enterprises’ financing constraints. The mechanism test shows that state equity participation reduces private enterprises’ external capital demand by alleviating competitive pressure, thus alleviating their financing constraints. Specifically, state equity participation significantly reduces obstacles to the court and tax systems. Second, because of the asymmetry of financial information and the bias of private enterprises towards financial institutions, the external financing demand generated by competitive pressure does not evolve into the behavior of external financing applications. Third, the heterogeneity analysis shows that state equity participation has a more significant mitigation effect on the financing constraints of young, nongroup, and non-listed enterprises. The policy implications of this paper are as follows. Firstly, the government should attach great importance to the financing constraints of private enterprises and actively guide the transfer of financial resources to private enterprises. Secondly, the government should encourage financial institutions to impart financing knowledge to private enterprises and enhance their understanding of financial information. Finally, the government should actively explore more neutral preferential policies to create a favorable competitive environment for private enterprises.

The generalizability of the results is limited by the lack of indicators of competitive pressure for Chinese main board listed companies. Another limitation of this paper is that it only explores financing constraints from the perspective of state participation and lacks attention to other politically relevant indicators, such as the political background of executives. In the future, we will continue to further investigate other political correlates of the above financing constraints.

## Supporting information

S1 Data (XLSX)
